# The Organophosphate Chlorpyrifos Interferes with the Responses to 17β-Estradiol in the Digestive Gland of the Marine Mussel *Mytilus galloprovincialis*

**DOI:** 10.1371/journal.pone.0019803

**Published:** 2011-05-20

**Authors:** Laura Canesi, Alessandro Negri, Cristina Barmo, Mohamed Banni, Gabriella Gallo, Aldo Viarengo, Francesco Dondero

**Affiliations:** 1 Dipartimento di Biologia, Università di Genova, Genova, Italy; 2 Dipartimento di Scienze dell'Ambiente e della Vita, Università del Piemonte Orientale ‘Amedeo Avogadro’, Alessandria, Italy; 3 Laboratory of Biochemistry and Environmental Toxicology, ISA, Chott-Mariem, Sousse, Tunisia; University of Houston, United States of America

## Abstract

**Background:**

Many pesticides have been shown to act as endocrine disrupters. Although the potencies of currently used pesticides as hormone agonists/antagonists are low compared with those of natural ligands, their ability to act via multiple mechanisms might enhance the biological effect. The organophosphate Chlorpyrifos (CHP) has been shown to be weakly estrogenic and cause adverse neurodevelopmental effects in mammals. However, no information is available on the endocrine effects of CHP in aquatic organisms. In the digestive gland of the bivalve *Mytilus galloprovincialis*, a target tissue of both estrogens and pesticides, the possible effects of CHP on the responses to the natural estrogen 17β-estradiol (E_2_) were investigated.

**Methodology/Principal Findings:**

Mussels were exposed to CHP (4.5 mg/l, 72 hrs) and subsequently injected with E_2_ (6.75 ng/g dw). Responses were evaluated in CHP, E_2_ and CHP/E_2_ treatment groups at 24 h p.i. by a biomarker/transcriptomic approach. CHP and E_2_ induced additive, synergistic, and antagonistic effects on lysosomal biomarkers (lysosomal membrane stability, lysosome/cytoplasm volume ratio, lipofuscin and neutral lipid accumulation). Additive and synergistic effects were also observed on the expression of estrogen-responsive genes (GSTπ, catalase, 5-HTR) evaluated by RT-Q-PCR. The use of a 1.7K cDNA *Mytilus* microarray showed that CHP, E_2_ and CHP/E_2_, induced 81, 44, and 65 Differentially Expressed Genes (DEGs), respectively. 24 genes were exclusively shared between CHP and CHP/E_2_, only 2 genes between E_2_ and CHP/E_2_. Moreover, 36 genes were uniquely modulated by CHP/E_2_. Gene ontology annotation was used to elucidate the putative mechanisms involved in the responses elicited by different treatments.

**Conclusions:**

The results show complex interactions between CHP and E_2_ in the digestive gland, indicating that the combination of certain pesticides and hormones may give rise to unexpected effects at the molecular/cellular level. Overall, these data demonstrate that CHP can interfere with the mussel responses to natural estrogens.

## Introduction

Many endocrine-disrupting compounds (EDCs) so far identified are persistent organochlorine pesticides (e.g., DDT, methoxychlor, dieldrin) [1]. Compared to these, modern pesticides, such as most organophosphates, do not bioaccumulate and therefore they might not reach concentrations able to cause endocrine disruption in humans or wildlife. However, organophosphorous and carbamate pesticides and their residues are present in the environment, in food items and human tissues and fluids all over the world [2], [3]; some of these have been reported to possess endocrine-disrupting properties [2], [4]–[6].

The potencies of pesticides as estrogen agonists/antagonists and antiandrogens *in vitro* are low compared with those of natural ligands [7]. However, chemicals with similar estrogenic potencies *in vitro* sometimes show very different potencies *in vivo* [8]. Their ability to act via more than one mechanism might enhance the biological effect in the intact organism, since the final response will likely be determined by the interactions of all pathways implicated. In this view, the application of ecotoxicogenomics, that is the study of gene expression in either target or non-target organisms, represents a powerful tool to understand, and infer, the molecular/cellular mechanisms involved in responses to environmental toxicant exposure in various species [9], [10].

Among the organophosphate insecticides, Chlorpyrifos (CHP) (phosphorothionic acid *O*, *O*-diethyl *O*-[3,5,6-trichloro-2-pyridyl] ester) was first introduced into marketplace in 1965 and used in agriculture worldwide [11]. The primary target organ for CHP is the nervous system, due to the ability of the chlorpyrifos-oxon metabolite to inhibit acetylcholinesterase (AChE) activity [11], [12]. However, several studies identified putative neurodevelopmental mechanisms that are independent of cholinesterase inhibition [11], [13]–[16]. CHP has been shown to interfere with different components of cell signalling [17]–[20], and to affect oxidative stress parameters in the developing brain, leading to shifts in expression and function of antioxidant genes [21], [22]. Beside brain defects, genital defects including undescended testes, microphallus, and fused labia were also reported [4], [5], [23]. *In vitro*, CHP showed a weak estrogenic activity in estrogenicity assays, and no significant effects on the response induced by 17β-estradiol were observed [7]. CHP also showed a weak increasing effect on the basal ERβ mRNA level in MCF-7 cells [24].

CHP is known to pose acute and chronic risks to many non-target wildlife [3], [6], [12], [25]. In terrestrial snails, long-term exposure to CHP induced lysosomal membrane destabilisation and increased AMPc (Cyclic Adenosine Monophosphate) levels in the digestive gland [26]. In the zebrafish, CHP did not lead to developmental alterations but induced the Hsp70 response as well as histopathological damage [27]. Bioconcentration of CHP has been investigated in bivalves [28], [29]. CHP significantly reduced AChE activity in both freshwater (*Amblema plicata*) and marine (*Mytilus galloprovincialis*) species [30], [31]. In the digestive gland of *M. galloprovincialis*, short term exposure (72 h) to low µM concentrations of CHP affected lysosomal biomarkers and gene expression [31]. In this species, the digestive gland, a tissue that plays a key role in metabolism and nutrient distribution to the gonad during gametogenesis, represents a target for the action of the natural estrogen 17β-estradiol (E_2_), as well as for estrogenic chemicals, both individually [32], [33] and in mixtures [34]. In particular, administration of estrogens by injection into the circulation significantly affected lysosomal biomarkers, antioxidant enzyme activities and gene expression, with both common and distinct effects of individual estrogens and mixtures [32]–[34].

In this work the possible effects of pre-exposure to CHP on the responses to E_2_ were evaluated in the digestive gland of *M. galloprovincialis*. Mussels were exposed to CHP (4.5 mg/l/animal) or vehicle for 72 hrs, subsequently injected with E_2_, and samples collected at 24 hr post-injection. Lysosomal biomarkers were evaluated and expression of individual genes was determined by RT-Q-PCR. Moreover, molecular responses to CHP-, E_2_- and CHP/E_2_-exposure were investigated by a transcriptomic approach utilizing a cDNA microarray developed for *M. galloprovincialis* (MytArray V 1.1) [31], [35]. The results indicate that in mussel digestive gland CHP interferes with the responses to the natural estrogen E_2_.

## Results

### Effects of CHP, E_2_ and CHP/E_2_ on lysosomal biomarkers

**Figure 1:**
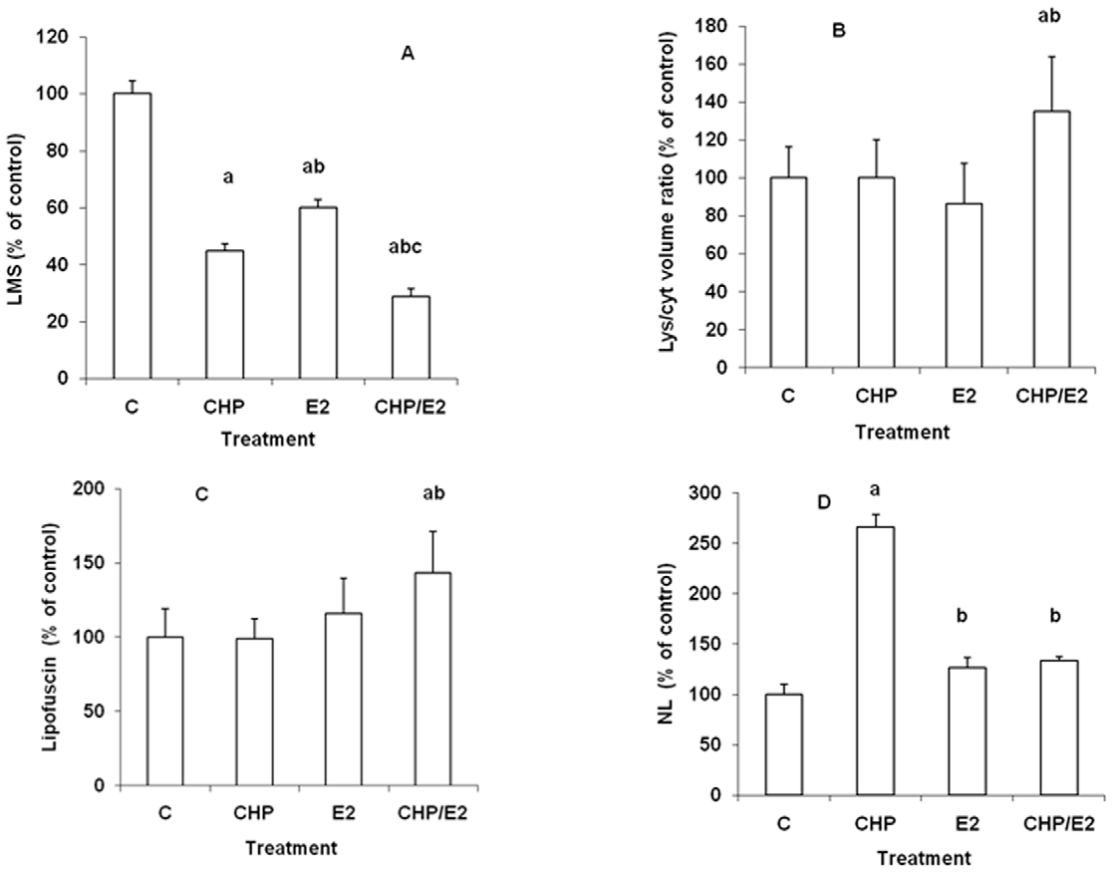
Effect of exposure to CHP, E_2_, or CHP/E_2_ on lysosomal parameters in *Mytilus galloprovincialis* digestive gland. Mussels were exposed for 72 hrs to CHP (4.5 mg/l ASW/animal) or vehicle (0.02% DMSO) and then injected with E_2_ (6.75 ng/g dw) or vehicle (0.05% ethanol) and tissues sampled 24 hrs post-injection. C = DMSO/EtOH. A) Lysosomal membrane stability (LMS); B) Lysosome/cytoplasm volume ratio; C) Lysosomal lipofuscin accumulation; D) Lysosomal Neutral Lipid accumulation. Data, expressed as % values with respect to controls, representing the mean±SD (n = 10), were analysed by ANOVA + Tukey's post test. a: all treatments *vs* C, P≤0.001; b: E_2_ *vs* CHPs,  = P≤0.001; c: CHP/E_2_ *vs* E_2_ and CHP = P≤0.001. b: CHP/E_2_ *vs* C and CHP  = P≤0.01; b: CHP/E_2_ *vs* E_2_ = P≤0.001. c: CHP/E_2_ *vs* C and CHP  = P≤0.001; b: CHP/E_2_ *vs* E_2_ = P≤0.05. d: CHP *vs* C, E_2_ and CHP/E_2_ = P≤0.001; b: E_2_ and CHP/E_2_ vs C = P≤0.05.

The effects of different exposure conditions (CHP, E_2_ and CHP/E_2_) on digestive gland lysosomal biomarkers were first evaluated and the results are reported in Fig. 1. As shown in Fig. 1A, CHP induced a significant decrease in lysosomal membrane stability-LMS (about −55% with respect to controls); a smaller effect was observed with E_2_ (−40%). Pre-exposure to CHP followed by E_2_ injection resulted in stronger lysosomal destabilisation (−71%). Representative images of the effects of differerent experimental conditions on LMS, evaluated as latency of the lysosomal N-acetyl-β-hexosaminidase activity, are reported in Fig. S1. The lysosome/cytoplasm volume ratio was unaffected by either individual treatment, whereas a significant increase was observed in CHP/E_2_ samples (+35% with respect to controls) (Fig. 1B). Similarly, neither CHP or E_2_ alone induced accumulation of lipofuscin, whereas a significant increase was observed in CHP/E_2_-treated mussels (+43% with respect to controls) (Fig. 1C). CHP induced a significant increase in neutral lipid (NL) content (up to +160% with respect to controls); a smaller effect was observed in response to E_2_ (+27%). In CHP/E_2_ treated mussels, the level of NLs was similar to that recorded in E_2_-injected mussels (+33% with respect to controls).

Neither vehicle (DMSO or Ethanol, alone or in combination) significantly affected lysosomal parameters in the digestive gland of mussels with respect to untreated mussels (not shown).

### Effects of CHP, E_2_ and CHP/E_2_ on expression of individual genes by RT-Q-PCR

**Figure 2:**
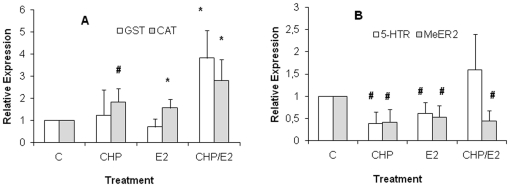
Effects of CHP, E_2_, or CHP/E_2_ on expression of estrogen-responsive genes in *Mytilus* evaluated by RT-Q-PCR. A) GST-π (GSH transferase) (AF527010) and catalase (AY743716); B) 5-HTR (*M. edulis* 5-hydroxytryptamine receptor) (AB526218) and MeER2 (*M. edulis* Estrogen Receptor 2 isoform) (AB257133). Gene expression was determined by quantitative RT-PCR as described in methods. The Relative Expression Software Tool (REST) [61] was used to calculate group means by means of the delta-delta Ct method adjusted for PCR efficiency using a 18S ribosomal target as reference gene [60] and data are reported as relative expression with respect to the control sample (DMSO/EtOH). Data are the mean±SD obtained from at least 4 independent RNA samples in triplicate.* = P≤0.05 Mann-Whitney U test.

The expression of genes whose transcription was shown to be modulated by individual estrogens or mixtures of estrogenic chemicals in *Mytilus* tissues [32]–[34], [36] was first evaluated by RT-Q-PCR through the sybr green I chemistry as previously described [37], and the results are reported in Fig. 2. These include genes involved in biotransformation and antioxidant defence (GST-π, catalase) and estrogen and serotonin (5-Hydroxy Tryptamine) receptors (*Mytilus* Estrogen Receptor MeER2 and 5-HT receptor), whose annotated sequences (see Table S1) were not included in the MytArray. CHP and E_2_ alone did not significantly affect the expression of GST-π (Fig. 2A); however, a large, significant increase in GST-π transcription was observed in CHP/E_2_ treated mussels (up to about 4-folds with respect to controls, P≤0.05). CHP and E_2_ alone induced a significant increase in transcription of catalase (Fig. 2B); an additive effect was observed in the CHP/E_2_ group (up to a 3-fold increase with respect to controls; P≤0.05). Moreover, both CHP and E_2_ alone induced a significant decrease in transcription of the 5-HTR; such down-regulation was not observed in the CHP/E_2_ group (Fig. 2C). On the other hand, transcription of the MeER2 receptors was similarly down-regulated in all exposure groups (Fig. 2D).

Neither vehicle (DMSO or Ethanol, alone or in combination) did significantly affect the expression of the genes considered in this study in the digestive gland of mussels (not shown).

### Transcriptomic analysis

**Figure 3:**
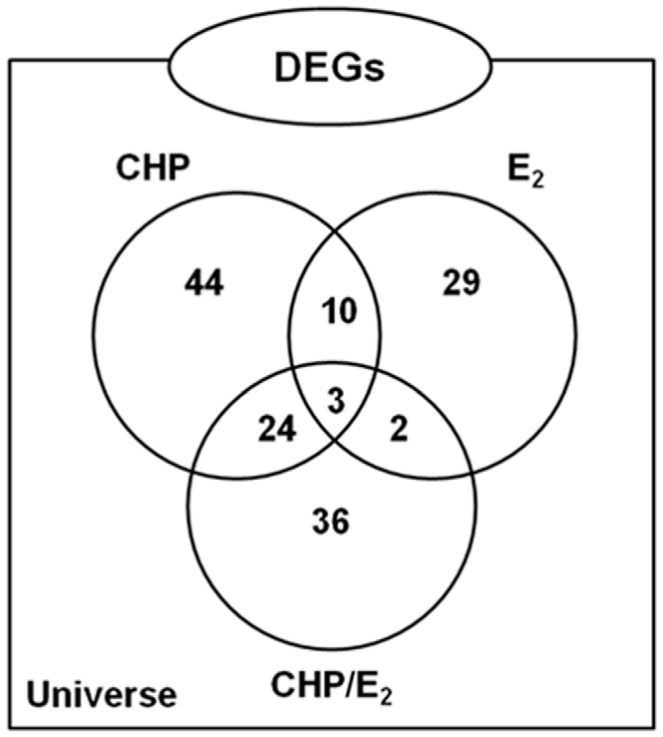
Venn diagram representation of gene expression patterns. The diagram clearly depicted that only two of the five overlapping genes were specifically shared between E_2_ and CHP/E_2_: AJ625117 with no annotation, and AJ516728, a putative dermatopontin. Data used to generate the Venn-diagram were obtained from microarray analysis (Table S1).

**Figure 4:**
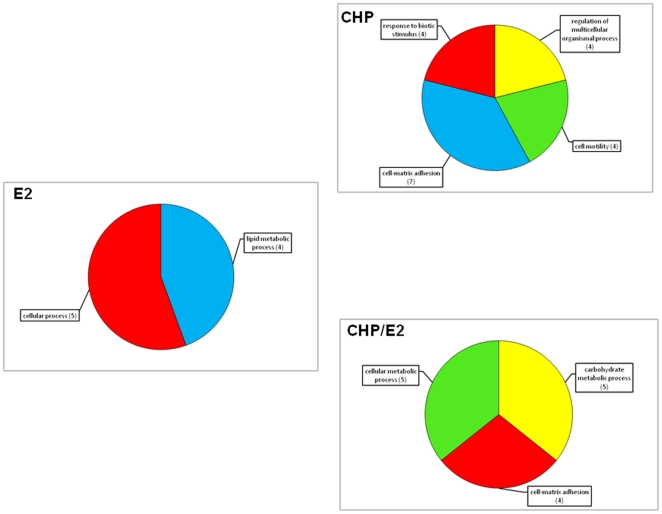
Functional genomics analysis: multi-level GO pie charts. The GO terms (biological processes) associated with the mussel sequences present in the array that resulted enriched by each treatment are reported (hypergeometric statistics, *p*<0.05). Due to the hierarchical structure of the GO tree, only the lowest nodes with at least four associated sequences were depicted. Additional information is given in Table S2.

**Figure 5:**
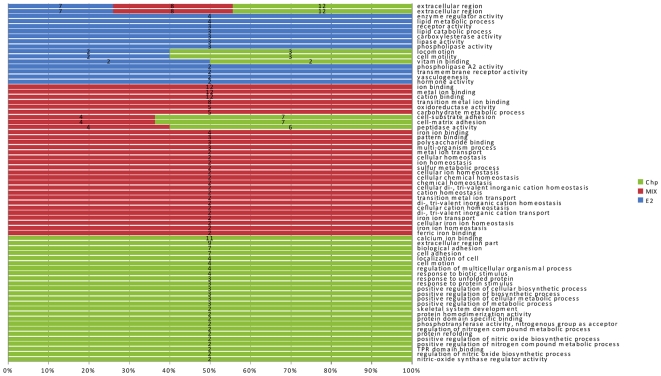
Functional genomics analysis: GO bar chart. GO terms (biological processes, molecular functions and cellular components) were obtained from a hypergeometric statistics (P<0.05) comparing the distribution of GO terms from each gene list with that obtained from the whole microarray catalogue. Bar length represents the relative frequency (%) of a GO term in each analyzed condition. Absolute frequencies of GO terms are also reported. Only GO terms with at least two associated genes were considered.

To get more clues on the molecular effects of E_2_ and the possible interference of pre-exposure with CHP with the responses to the hormone, we carried out a trascriptomic analysis on digestive gland RNA samples by means of the MytArray V1.1 1.7 K cDNA chip [31], [35] (Table S1). Dual color hybridisation microarray analysis unveiled a total of 148 differentially expressed genes (DEGs) in at least one out the three analyzed conditions (CHP, E_2_ and CHP/E_2_) (Fig. 3 and Table S1). CHP alone elicited the highest molecular responses displaying 81 DEGs of which 73% (n = 59) were up-regulated (Table S1). In E_2_-treated mussels, microarray analysis displayed 44 DEGs with 29 up-regulations (66%), while the CHP/E_2_ group showed 65 DEGs, mostly up-regulated (53 genes, 81%). About 41% of DEGs (n = 27) found in the CHP/E_2_ group overlapped with those modulated by CHP, whereas only the 8% (5 genes) was shared with E_2_. The expression of another set of 36 DEGs was modulated only in CHP/E_2_ samples (Fig. 3). A functional genomic analysis based on Gene Ontology term distribution was carried out to unravel the biological processes and molecular functions over-represented in each DEG list. To this aim, each set of GO (Gene Ontology) terms associated with a gene list was filtered against the reference set of GO terms associated with the whole array-sequence catalog by means of a hypergeometric statistics (Fisher's exact test, P<0.05). These results are summarized in Fig. 4 and Fig. 5 (see also Table S2). Moreover, to infer virtual biological interactions elicited by the joint action of the pesticide and E_2_, we used the same statistical approach to highlight GO terms that were over-represented in the E_2_ gene list with respect to the CHP/E_2_ group (Table 1).

**Table 1:** E_2_ specific GO terms.

GO Term	Name	p-Value	**#** in test group	**#** in reference group	**#** non annot test	**#** non annot reference group
GO:0044425	membrane part	0.03	4	0	18	31
GO:0004871	Signal transducer activity	0.03	4	0	18	31
GO:0060089	molecular transducer activity	0.03	4	0	18	31
GO:0004872	receptor activity	0.03	4	0	18	31
GO:0016020	membrane	0.04	5	1	17	30

Hypergeometric statistics was used to compare the GO term distribution in the E_2_ gene list *vs* the CHP/E_2_ group to identify processes characteristic of the hormone. **# in test group**: number of genes associated with each respective GO term into the test group (E_2_); **# in reference group**: number of genes associated to each respective GO terms into the reference group (CHP/E_2_); **# non annot test**: number of genes not annotated into the test group (E_2_); **non annot in reference group**: number of genes not annotated into the reference group (CHP/E_2_).

**Figure 6:**
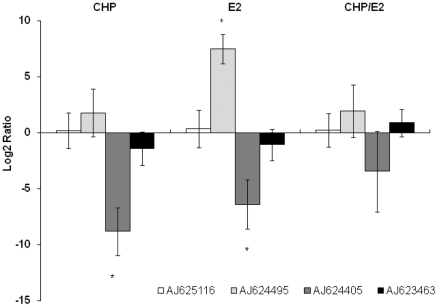
RT-Q-PCR analysis. Actin (AJ625116); GM2-activator protein: GM2-AP (AJ624495), GM2-AP (AJ624405); hexosaminidase (AJ623463). The actin gene analyzed by RT-Q-PCR, which showed no expression changes from microarray analysis, was included in this survey as a confirmation of the normalization process based on the expression of the 18S rRNA. Log2 group mean relative expression levels with respect to control (DMSO/ETOH) ±SD (n = 4) are reported; * = p<0.05 Mann-Whitney U test.

RT-Q-PCR analysis was further carried out to confirm the expression of selected genes: two homologue GM2-Activator Protein (AP) genes (AJ624495, AJ624405), hexosaminidase (AJ623463) and actin (AJ625116) (Fig. 6). Vehicles (DMSO or Ethanol, alone or in combination) did not affect the expression of the genes considered in this study (data not shown). As shown in Fig. 6, GM2-AP genes showed two opposite expression trends characterized, in general, by an up-regulation of AJ624495 and down-regulation of the cognate sequence AJ624405. The expression of the latter gene was significantly affected by CHP and E_2_ alone, whereas that of AJ624495 was significantly increased only in response to the hormone. By contrast, hexosaminidase and actin expression patterns were not significantly affected in any experimental condition. The pattern of GM2 AJ624495, as well those of hexosaminidase and actin obtained from RT-Q-PCR fitted with the outcome of microarray data (Table S1).

## Discussion

In this work, the hypothesis that in *M. galloprovincialis* digestive gland pre-exposure to CHP may interfere with the molecular and cellular responses to the natural hormone E_2_ was investigated. To this aim, a combination of core biomarkers -i.e lysosomal parameters- and gene expression/functional genomic techniques was utilised. Moreover, the present study represents the first investigation on the effects of natural estrogens in a molluscan species based on a transcriptomic approach. Both CHP and E_2_ individually have been previously shown to induce dose-dependent effects on different biomarkers and gene expression in mussel digestive gland [31], [32]. The results here presented demonstrate that pre-exposure of mussels to sublethal concentrations of CHP affects the responses to E_2._

The CHP exposure dose corresponded to the EC_50_ values previously obtained in CHP toxicity assessment in the same experimental conditions, utilising LMS data, showing a clear dose-response trend with exposure [31]. Under these conditions, about 40% inhibition of digestive gland acetylcholinesterase activity, evaluated as a specific biomarker of exposure to the organophosphate pesticide, was observed [38].

The E_2_ injection protocol was utilized instead of estrogen addition in artificial sea water-ASW since this protocol of exposure to E_2_ in the physiological nM concentration range allowed the evaluation of the effects of the hormone on both digestive gland and immune function in *M. galloprovincialis* [32], [33], [39], probably bypassing the *in vivo* homeostatic control of E_2_ levels by steroid esterification in the tissues [40]. The effects of E_2_ were apparently mediated by non-genomic mechanisms [39], [41]. In the digestive gland, responses of lysosomal biomarkers to E_2_ injection indicated dose-dependent decrease in LMS and increase in NL accumulation, with no effect on lipofuscin accumulation [32].

### Effects of CHP, E_2_ and CHP/E_2_ on lysosomal biomarkers and individual gene expression

Both CHP and E_2_ alone induced lysosomal destabilisation and a larger effect was recorded in CHP/E_2_-exposed mussels. On the other hand, although neither treatment significantly affected the lysosome/cytoplasm volume ratio, or lipofuscin accumulation, in CHP/E_2_ exposed mussels a significant increase in both parameters was observed. CHP induced strong NL accumulation as already reported [31], whereas a smaller effect was observed with E_2_ [32]; however, the effect of CHP was dramatically reduced in E_2_-injected animals. These data indicate that the organophosphate pesticide and the natural estrogen can exert not only additive, but also synergistic and antagonistic effects on lysosomal biomarkers. Interactive effects of CHP and E_2_ were also observed on the expression of individual genes. In mussel digestive gland, CHP and E_2_ induced a synergistic effect on the GST-π mRNA levels, the main GST isoform expressed in mussel tissues [42], whereas an additive effect was observed on catalase up-regulation. In differentiating PC12 cells, a well-established neurodevelopmental model, CHP elicited significant up-regulation of catalase and of various GSTs [22].

In mammals, recent studies showed that not only acetylcholine systems but also developing serotonin (5HT) systems may be sensitive to organophosphates, with exposure producing long-term changes in 5HT synaptic function and associated behaviors (see [16] and references quoted therein). Our data indicate that in mussel digestive gland CHP induced down-regulation of the 5-HT Receptor-; a similar effect was elicited by E_2_, as previously described in the mantle [36], whereas no significant effects were observed in CHP/E_2_ treated mussels.

In mammalian cells, organophosphorous pesticides also possess the ability to interfere with the ERα and ERβ mRNA steady state levels [24], according to the reported weak estrogenic properties of the pesticide [7]. Both CHP and E_2_ induced downregulation of the MeER2 gene in mussel digestive gland; however, no differences were observed in mussel exposed to CHP/E_2_ with respect to individual treatments. Although increases in MeER2 expression were found in *Mytilus* tissues in response to E_2_ [32], [43], decreases in MeER2 mRNA levels in female digestive glands (this study), as well as in the gonad of mature females observed in response E_2_ [43] suggest that E_2_-induced receptor downregulation may occur in female tissues at certain stages of gametogenesis.

### Evidence for seasonal dependent effects in the response to Chlorpyrifos

In marine bivalves, and in particular in *Mytilus spp.*, seasonal changes have long been described in different parameters, from the molecular to the organism level, in relation to differences in both abiotic and biotic factors, such as temperature, food availability and reproductive stage [44]. These in turn have been shown to affect the responses to contaminant exposure [44], [45]. A clear temporal pattern in gene expression profiles has been recently described in the tissues of a natural mussel population of *M. galloprovincialis* sampled over an annual cycle, according to physiological changes in metabolic processes related to the reproductive stage [46]. In the digestive gland of female mussels largest differences were observed between January and June-July, but also between March (spawning stage) with respect to October (developing stage). These data were in line with the key features of the annual reproductive cycle of *Mytilus spp*.

The effects of CHP exposure on mussel digestive gland have been recently characterized by a combination of a biomarker/transcriptomic approach, utilising mussels sampled in March, during the mature stage of the gonad [31]. In the present work, experiments were carried out in mussels collected in fall (October), when most female individuals were in the immature-developing stage (not shown). In general, the results of lysosomal biomarkers displayed similar outcomes with respect to LMS and NL accumulation in the two experiments; on the other hand, the lysosome/cytoplasm ratio was affected by CHP exposure in March [31], but not in October [this work]. Since pollutant-induced increase in lysosome activity involves autophagic processes, reduction of the cytoplasm of the cells and consequent adverse effects at the tissue level [47], these data indicate the occurrence of a less severe stress syndrome induced by the pesticide in mussels sampled in fall.

This observation is supported by data obtained at the molecular level, where more marked seasonal differences in the response to CHP were observed. The number of DEGs found in the present study was twice as high as that previously observed (81 vs 43), with only 6 genes in common: the two mam domain containing 2 (AJ624363; AJ624502), ferritin (AJ625268); heat shock protein 90 (AJ625974), a mucin-like protein (AJ624419) and an unknown sequence (AJ625629). Moreover, the mRNA level of a 3′-Phosphoadenosine-5′-phosphosulfate (PAPS) synthetase gene (AJ624309), a coenzyme in sulphotransferase reactions in phase II of xenobiotic biotransformation, sharply increased in response to CHP only in the digestive gland of animals samples in fall (Table S1). The CHP-induced up-regulation of genes involved in carbohydrate metabolism, in particular those related to chitinase activities, observed in mussels sampled in March [31], were no longer observed in mussels sampled in October (this study). Also relative abundances of mRNA for the two GM2-AP genes, although showing the same trend in response to CHP, were very different. Overall, these data further support the hypothesis that seasonal changes in the physiological status can significantly affect the response of mussel tissues to contaminants, not only at the biochemical level, but also at the transcriptional level.

### Effects of E_2_ on transcriptomics

Administration of E_2_ by injection into the mussel vascular system resulted in the modulation of 44 genes (about 2.5% of sequences present in the array), 23 of which bore a functional annotation (GO terms) assigned by the Blast2GO system [48]. Functional genomics indicated that about 50% of the annotated DEGs found in response to E_2_ injection are involved in primary metabolic processes (n = 12), such as lipid catabolism (Fig. 4, 5). Among these, two sequences coded for phospholipase A (PLA) (Table S2). E_2_ also induced an increase in the mRNA level of calmodulin gene, which might indicate effects on Ca^2+^ homeostasis. E_2_ was previously shown to induce an intracellular [Ca^2+^] rise in mussel hemocytes *in vitro* [41], [49]. Moreover, in these cells, activation of Ca^2+^-dependent PLA_2_ was involved in mediating E_2_-induced lysosomal membrane destabilization [49]. The results obtained *in vivo* on digestive gland lysosomal biomarkers support the hypothesis of a similar mechanism driven by E_2_ also in the digestive gland cells, possibly involving Ca^2+^ homeostasis and PLA_2_ in modulation of gene expression.

Another gene involved in lysosomal lipid metabolism, whose expression was modulated by E_2_, coded for the ganglioside GM2-Activator Protein (GM2-AP) (AJ624495). The GM2-activator is a glycoprotein required for the *in vivo* degradation of ganglioside GM2 by hexosaminidase A [50]. Indeed, two highly homologue GM2-AP genes are represented in the Myt-array V1.1 and therefore the correct expression pattern was investigated by Taqman multiplexed RT-Q-PCR (Fig. 2). This analysis not only confirmed the over-expression of the AJ624495 GM2-AP sequence in E_2_-treated samples, but also showed a large decrease in the cognate mRNA level (AJ624405) (Fig. 6). The discrepancy between microarray and RT-Q-PCR data was probably due to the high sequence homology of GM2-AP genes which could not be discriminated merely by the use of a hybridization based assay. Previous studies carried out by our research group indicated that such peculiar expression trend in GM2-AP sequences was found in response to various toxic chemicals and that it might be related to a lysosomal lipidosis syndrome [31]. However, further investigation is required to elucidate the role of such genes in lysosomal lipid homeostasis of mussel digestive gland.

In E_2_-treated samples transcriptomics and further GO terms analysis based on functional genomics also underlined the occurrence of virtual biological processes and molecular functions typical of a hormone-induced response. Indeed, specific GO terms such as “hormone response”, “receptor activity”, “vasculogenesis” and “heart development” were over-represented in the E_2_ DEG list (Fig. 5). Linked to the GO term “hormone response” are the mucin-like genes (AJ624419; AJ516390), that were over-expressed in response to E_2_, and the proto-oncogene myc, that was instead down-regulated (Table S1). Mucin genes are known to be up-regulated by estradiol and the secretion of such proteins is known to increase in a variety of normal and tumor mammalian cells [51], [52]. Other genes associated with the GO terms vasculogenesis and heart development might be implicated in some developmental processes of smooth muscle cells. Among genes bearing those features, we found two mam-domain containing-2 proteins (AJ624363; AJ624502) that are involved in angiogenesis [53], and an integrin beta-1 gene (fibronectin receptor beta, AJ626301) putatively implicated in myogenesis [54]. E_2_ injection in mussels also elicited the over-expression of several other muscle proteins such as tropomyosin (AJ625392), paramyosin (AJ624823) and catchin (AJ625393), a variant of myosin (Table S1).

### Chlorpyrifos pre-exposure abolished the E_2_ specific molecular fingerprint

Our data show that mussel pre-exposure to sublethal concentrations of CHP affected the transcriptomic fingerprint obtained in response to E_2_ alone. This was clearly depicted by the fact that only two genes, dermatopontin (AJ516728) and an unknown sequence (AJ625117), were specifically in common (3.1%) between the E_2_ and CHP/E_2_ DEG lists (Fig. 3). Conversely, much more similarity was found between CHP and CHP/E_2_ treatments, as these two conditions displayed 24 (37%) identical DEGs (Fig. 3; Table S1). Furthermore, functional genomic analysis showed that a relevant part of this common set of sequences were found associated with the same over-represented GO terms. These findings indicate that CHP pre-exposure could virtually influence functional responses to E_2_ abolishing the estradiol-like molecular responses (Fig 4, 5; Table S2). It is worth noting that most sequences obtained for the CHP/E_2_ group by means of microarray analysis represented unique genes (Fig. 3; Table S1), that might give rise to unique molecular functions and/or virtual biological processes (Fig. 5). These data support the hypothesis that contaminants like pesticides can show novel, unpredictable modes of action when interfering with natural/endogenous compounds such as hormones. The results obtained on the expression of individual gene sequences by RT-Q-PCR also displayed this trend (Fig. 2). These effects were also reflected at the cellular/tissue level, as indicated by biomarker data showing interactive outcomes at lysosomal level.

### Conclusions

The results presented in this work indicate that CHP exposure affects the responses of mussel digestive gland to the natural estrogen E_2_. In mussel cells, E_2_ has been shown to activate both Ca^2+^- and kinase mediated transduction pathways [38], [40]. In particular, E_2_ activates PKC (protein kinase C) and MAPK (Mitogen activated protein kinase) signaling, leading to increased phosphorylation of different transcription factors, including STAT members (Signal Transducers and Activators of Transcription) and CREB (Cyclic AMP Responsive Element Binding Protein) [39], [41]. In the digestive gland, both genomic and non-genomic modes of action involving ER-like receptors, as well as receptor-independent mechanisms, may participate in mediating the effects of E_2_. In this tissue, E_2_ was shown to modulate the lysosomal function as well as lipid and carbohydrate metabolism [33]; the results of microarray data confirm that E_2_ can affect the expression of genes related to the lysosomal function and lipid metabolism, supporting the hypothesis that estrogens may also play an indirect role in gametogenesis, by affecting nutrient metabolism and accumulation. As to the possible mechanisms by which CHP could interfere with estrogen action, non anti-cholinesterase mechanisms of CHP toxicity involved altered PKC, MAPK and Ca^2+^-AMPc signaling [19], [20], [55], [56]. Overall, our results support the effectiveness of a biomarkers/genomics approach to assess the effects of 17β-estradiol in the digestive gland of the marine mussel *M. galloprovincialis*, and demonstrate that sublethal amounts of an organophosphate pesticide, such as CHP, are able to interfere with the responses to natural estrogens. In this light, our data also indicate that CHP can act as an endocrine disrupter in the digestive gland of mussels.

## Materials and Methods

### Animals and treatments

Mussels (*Mytilus galloprovincialis* Lam.) (5–6 cm length) were obtained from a mussel farm in Cesenatico (RN, Italy) in October 2006, and transferred to aquaria with recirculating aerated seawater collected offshore, at a density of 1 animal/L. After an acclimation of 6 days at 16°C, groups of mussels were kept in static tanks (1 animal/L seawater) and exposed to different experimental conditions. Groups of mussels (4 of 15 animals each) were exposed for 72 h to CHP (4,5 mg/l ASW) from a stock solution in DMSO. The same number of control animals were added with the same amount of vehicle (final DMSO concentration 0.02%). CHP was administered every day, together with a commercial algal preparation (Liquifry, Interpret Ltd., Dorking, Surrey, UK) and seawater renewed every two days. After exposure, half of control and CHP-exposed mussels were injected into the posterior adductor muscle with 50 µl of an E_2_ solution (0.5 µM) (from a 10 mM stock solution in ethanol diluted in ASW), using a sterile 0.1 ml syringe as previously described [32], [39], [41]. The remaining mussels were injected with 50 µl of a solution of ASW containing an equal amount of ethanol (0.05%). After injection, mussels were kept in separate tanks in clean ASW and tissues sampled after 24 h.

The CHP concentration used corresponded to the EC_50_ calculated from data on digestive gland LMS, previously utilized as the guide biomarker in CHP toxicity assessment [31]. The nominal E_2_ concentration (6,75 ng/g dw, 25 pmoles/ml hemolymph) was chosen on the basis of previous data on the effects of E_2_ exposure on mussels in similar experimental conditions [32], [39], [41], on the circulating levels of free E_2_ in the hemolymph (about 3 pmoles/ml), and taking into account an average dry weight of whole animal soft tissues of about 1 g.

In all experiments female individuals -screened by microscopic inspection of Toluidine blue stained cross sections (2 µm) of resin embedded mantle biopsies- were used for subsequent analyses. Most individuals (about 87%) were in the I-II stage, indicating immature-developing gonad, with small percentages in the III or IV stage (ripe, spawning). After treatments, digestive glands were rapidly removed, frozen in liquid N_2_ and stored at −80°C. For transcriptomics, tissues were kept at −20°C in a RNA preserving solution (RNA Later, Sigma-Aldrich); for histochemistry, tissues were mounted on aluminum chucks and frozen in super-cooled n-hexane and stored at −80°C.

### Lysosomal biomarkers

Lysosomal membrane stability-LMS, lysosomal neutral lipid (NL) and lipofuscin (LF) content, and lysosomal/cytoplasm volume ratio, were evaluated in duplicate cryostat sections of 5 digestive glands according to [57]. Sections (10 µm) were cut with a Leica cryostat, flash-dried by transferring them to room temperature, and then stained for *N*-acetyl-β-hexosaminidase activity [58]. LMS was evaluated by assessment of latency of lysosomal N-acetyl-β-hexosaminidase (min). Representative images of lysosomal staining in different experimental conditions are reported in Fig. S1. Lysosomal staining intensity was obtained by means of an inverted Axiovert microscope (Zeiss) at 400×magnification, connected to a digital camera (Axiocam, Zeiss). Digital image analysis was carried out using the Scion Image software package (Scion Corp. Inc.) from 8-bit gray scale images. Data were expressed as percent LMS values with respect to controls.

Neutral lipid content was evaluated in cryostat sections of digestive glands fixed in calcium-formaldehyde (2% Ca-acetate (w/v), 10% formaldehyde (v/v)) for 15 min at 4°C, followed by a rinsing step with de-ionised water, and incubation with 60% triethylphosphate (TEP) for 3 min. The sections were then stained with Oil Red-O (1% in 60% TEP) for 30 s, rinsed with de-ionised water, and mounted in 20% (v/v) glycerol. Lipofuscin content was determined using the Schmorl reaction on cryostat sections fixed in calcium-formaldehyde and rinsed with de-ionised water, as described for the neutral lipid assay, followed by a 5 min incubation step with 1% Fe_2_Cl_3_, 1% potassium ferrocyanide in a 3∶1 ratio [57]. The sections were rinsed with 1% acetic acid and mounted in 20% (v/v) glycerol. Neutral lipid and lipofuscin content were quantified by digital image analysis of stained sections, as described for the LMS assay.

Lysosome/cytoplasm volume ratio was determined on the same sections used for LMS determination by evaluating the cytoplasmic and lysosomal areas [58], [59].

### Quantitative RT-PCR analysis

>Total RNA was extracted from pools of 6 digestive gland pieces using the TRI-Reagent (Sigma-Aldrich). RNA was further purified by precipitation in the presence of 1.5 M LiCl. The quality of each RNA preparation was verified both by UV spectroscopy and TBE agarose gel electrophoresis, in the presence of formamide as previously described [60]. Expression levels of GSTπ [GeneBank: AF527010], Catalase [GeneBank: AY743716], serotonin (5-HT) receptor [GeneBank: AB526218] and *Mytilus* estrogen receptor 2 (MeER2) [GeneBank:AB257133] were evaluated as previously described [36]. Aliquots of 1 µg RNA were reverse-transcribed into cDNA using 200 units RevertAid H Minus M-MuLV Reverse Transcriptase (Fermentas Italy, M-Medical, Milan), in presence of 200 ng of Random Examers (Fermentas), 1 mM dNTPs (Fermentas) at 42°C for 60 min in a reaction volume of 20 µl. The cDNA was used to amplify the genes of interest using a Chromo 4™ System real-time PCR apparatus (Biorad Italy, Segrate, Milan). Proper aliquots of the RT mixture were diluted to a final volume of 20 µl in presence of iTaq SYBR Green Supermix with Rox (Biorad) and 0.25 µM of each specific primer pairs (TibMolBiol, Genoa, Italy). The primer pairs used and their accession numbers are shown in Table S1. Thermal protocol consisted of 3 min initial denaturation at 95°C followed by 40 cycles: 15 s at 95°C, 30 s at 55°C (30 s at 54°C for MeER2; 30 s at 60°C for 5-HT Receptor), 20 s at 72°C. A melting curve of PCR products (55–94°C) was also performed to ensure the presence of artifacts. Expression level of 18S did not change in samples obtained from different experimental conditions (data not shown). Therefore, expression of the genes of interest was normalized using the expression levels of 18S as a reference [37]. Relative expression of target genes in comparison with that of the 18S mRNA reference gene was conducted following the comparative Ct threshold method [61] using the Biorad software tool Genex-Gene Expression MacroTM [62]. The normalized expression was then expressed as relative quantity of mRNA (relative expression) with respect to the control sample. Data are the mean ±SD of at least 4 samples measured in triplicate.

For validation of microarray data, Multiplex TaqMan gene expression assay was used to assess the expression of actin [GeneBank:L33452], GM2-activator [GeneBank:AJ624495, GeneBank:AJ624405] and hexosaminidase [GeneBank:AJ623463] genes as described in [31].

### Microarray hybridization analysis

Competitive, dual color microarray hybridization analyses were performed on the same RNA samples used for RT-Q-PCR analysis following a common reference design in which each experimental condition was hybridized against the same reference condition, i.e. digestive gland tissue from vehicle treated animals. Four different biological replicates were used to analyze each condition. One replicate per array was used. Microarray analysis was performed using the MytArray platform [35] (V1.1) essentially as described in [60]. Pre-processing and differentially expressed genes were obtained by means of the R based package LIMMA [60], [63] through the implementation of empirical Bayes statistics. B>0, where B-statistics represents the log-odds that that gene is differentially expressed.

### Functional genomic analysis

Functional characterization of mussel genes present in the array was based on Gene Ontology annotation and it was carried out by means of the universal platform Blast2GO (B2GO) [48], using default parameters. GO term enrichment analysis was carried out through the implementation of a hypergeometric statistics (p<0.05).

MIAME compliant microarray data (including a detailed description of each hybridization experiment) were deposited in the Gene Expression Omnibus (GEO) database, with the superSeries unique identifier GSE26222.

## Supporting Information

Table S1 **Microarray gene expression profiles.** For each experimental condition (CHP, E_2_, CHP/E_2_) the embl gene ID (Gene) and the putative description assigned by means of the bioinformatic platform Blast2GO [48] are reported; M  =  log2 gene relative expression level; B  =  empirical Bayes log odd; Adj P  =  adjusted p value according to 64. A gene was considered differentially expressed when a B>0 value was obtained according to the empyrical Bayes B-statistics 65. B values lower that 0 are shown in red.
(PDF)

Table S2 **Supplementary information to** **Fig. 4** **.** Gene ID, gene description, expression trend of sequences reported in Fig. 4 are reported.
(PDF)

Figure S1 **Determination of Lysosomal membrane stability (LMS) by assessment of latent lysosomal N-acetyl-508β-hexosaminidase activity in cryostat sections of frozen mussel digestive gland as described in** [**58**] **.** Sections were pre-treated at pH 4.5 and 37°C for 3–40 minutes (3, 5, 10, 15, 20, 30, 40 minutes, respectively). Representative images of A =  Control DMSO/EtOH; B =  CHP; C =  E2; D =  CHP/E2, where maximal lysosomal staining intensity represents the labilization period. (Scale Bar  = 10 µm).
(TIF)
