# Supplementary material for: The Organophosphate Chlorpyrifos Interferes with the Responses to 17β-Estradiol in the Digestive Gland of the Marine Mussel Mytilus galloprovincialis
Source: PLoS One. 2011 May 20;6(5):e19803. doi: 10.1371/journal.pone.0019803 (PMC3098840; doi:10.1371/journal.pone.0019803)
Supplement: Table S1 — Microarray gene expression profiles. For each experimental condition (CHP, E2, CHP/E2) the embl gene ID (Gene) and the putative description assigned by means of the bioinformatic platform Blast2GO [48] are reported; M = log2 gene relative expression level; B = empirical Bayes log odd; Adj P = adjusted p value according to 64. A gene was considered differentially expressed when a B>0 value was obtained according to the empyrical Bayes B-statistics 65. B values lower that 0 are shown in red. (PDF) [file pone.0019803.s001.pdf]

Table S1. Microarray gene expression profiles

		CHP			E2			CHP/E2		
Gene	Description	M	B	Adj p	M	B	Adj p	M	B	Adj p
AJ624502	mam domain containing 2	-1.58	16.84	6.85E-09	-1.31	13.4	5.45E-07	0.09	-6.52	9.67E-01
AJ624363	mam domain containing 2	-1.88	19.62	5.51E-10	-1.26	12.19	1.02E-06	0.19	-6.56	9.90E-01
AJ624704	collagen typealpha 1	-1.32	11.48	5.05E-07	-1.03	9.46	1.27E-05	0.17	-6.53	9.74E-01
AJ624465	cg3355-isoform a [trypsin]	0.73	8.83	4.67E-06	0.79	8.15	3.78E-05	0.61	3.99	7.73E-04
AJ623339	---NA---	-1.32	14.79	4.31E-08	-0.82	7.85	4.18E-05	0.36	-5.72	5.70E-01
AJ623586	---NA---	0.29	-3.97	1.03E-01	0.70	5.98	2.19E-04	-0.03	-6.54	9.86E-01
AJ625393	catchin	0.03	-6.86	9.90E-01	0.6	5.95	2.19E-04	-0.09	-5.74	5.76E-01
AJ624667	cystathionine beta-synthase	0.7	9.4	2.99E-06	0.72	5.81	2.21E-04	0.56	2.77	1.30E-03
AJ626121	---NA---	0.48	-0.09	7.07E-03	0.77	5.32	3.30E-04	0.06	-6.36	8.65E-01
AJ516665	---NA---	-0.52	0.02	6.72E-03	-0.58	4.97	4.27E-04	0.01	-6.54	9.86E-01
AJ625392	tropomyosin	0.16	-6.66	8.15E-01	0.46	4.8	4.61E-04	0.33	-2.01	4.57E-02
AJ516728	dermatopontin	-0.13	-6.58	7.72E-01	-0.91	4.44	6.19E-04	-0.91	2.51	1.61E-03
AJ624823	paramyosin	0.22	-5.91	4.75E-01	0.43	4.15	7.70E-04	0.06	-6.54	9.86E-01
AJ516452	ependymin-related protein	1.61	6.01	4.42E-05	1.36	3.79	1.04E-03	0.64	-4.01	1.86E-01
AJ624419	mucin-like protein	0.63	3.2	4.79E-04	0.69	2.87	2.39E-03	0.13	-5.61	5.40E-01
AJ623565	---NA---	-1.44	-1.03	1.34E-02	-1.21	2.81	2.39E-03	-0.06	-6.44	9.08E-01
AJ516558	viral a-type inclusion	-0.11	-4.94	2.18E-01	1.4	2.81	2.39E-03	0.92	-2.1	4.86E-02
AJ516741	myc homolog	-0.07	-6.51	7.36E-01	-0.44	2.6	2.80E-03	-0.19	-5.01	3.55E-01
AJ623461	---NA---	0.43	-0.58	9.97E-03	0.47	2.5	2.94E-03	0.1	-6.53	9.74E-01
AJ625774	---NA---	0.21	-5.77	4.26E-01	0.75	2.39	3.14E-03	0.4	-4.46	2.51E-01
AJ625117	---NA---	0.05	-6.63	7.96E-01	0.49	2.27	3.37E-03	0.78	1.37	3.92E-03
AJ623364	Phospholipase A1 member i	-0.04	-6.65	8.02E-01	0.78	2.09	3.88E-03	0.34	-4.62	2.81E-01
AJ624368	scavenger receptor cysteine-richpartial	0.06	-6.51	7.32E-01	-1.11	1.97	4.21E-03	0.41	-3.71	1.49E-01

AJ625949	developmentally-regulated vdg3	0.56	0.32	5.64E-03	0.79	1.81	4.77E-03	0.36	-5.62	5.41E-01
AJ623567	---NA---	0.1	-5.26	2.79E-01	0.67	1.65	5.43E-03	0.37	-6.25	8.00E-01
AJ516720	vitelline coat lysin m7	-0.07	-6	5.02E-01	1.14	1.53	5.76E-03	0.45	-5.14	3.83E-01
AJ624059	calmodulin	0.63	1.51	2.08E-03	0.46	1.51	5.76E-03	-0.04	-6.56	9.98E-01
AJ516365	af101063_1intermediate filament filarin	-0.05	-6.76	8.90E-01	0.72	1.38	6.42E-03	0.14	-6.38	8.74E-01
AJ624693	kiaa0256 protein	0.67	0.72	4.07E-03	0.77	1.11	8.20E-03	1.08	2.01	2.42E-03
AJ624414	---NA---	0.49	-2.13	2.84E-02	0.64	1.05	8.42E-03	0.55	-3.32	1.13E-01
AJ624079	cytochrome c oxidase subunit iv	0.28	-2.06	2.67E-02	0.32	0.78	1.08E-02	-0.2	-3.99	1.83E-01
AJ624501	---NA---	-0.69	-5.51	3.41E-01	-1.08	0.7	1.09E-02	-0.19	-4.34	2.32E-01
AJ624908	---NA---	-0.56	-4.86	2.03E-01	-0.73	0.64	1.09E-02	-0.07	-5.11	3.79E-01
AJ624889	---NA---	-0.16	-6.72	8.57E-01	0.57	0.63	1.09E-02	0	-6.55	9.90E-01
AJ623571	---NA---	-0.34	-2.49	3.62E-02	-0.32	0.62	1.09E-02	0.02	-6.3	8.34E-01
AJ625974	heat shock protein 90	-0.35	4.17	2.11E-04	-0.32	0.62	1.09E-02	-0.26	-0.5	1.49E-02
AJ625292	cg7231-isoform c	-0.5	-6.32	6.36E-01	-1.09	0.6	1.09E-02	-0.18	-4.02	1.86E-01
AJ625312	---NA---	0.32	-6.85	9.73E-01	-0.98	0.53	1.15E-02	-0.13	-6.55	9.90E-01
AJ625670	---NA---	0.31	-3.34	6.56E-02	0.32	0.38	1.31E-02	-0.04	-6.05	7.12E-01
AJ624509	phospholipasegroup xiia	0.4	-5.94	4.82E-01	1.01	0.25	1.46E-02	0.34	-6.51	9.66E-01
AJ624495	gm2 ganglioside activator protein	1.04	-2.03	2.62E-02	1.25	0.18	1.52E-02	1.32	-1.12	2.28E-02
AJ623396	---NA---	0.37	-2.3	3.21E-02	0.67	0.12	1.57E-02	0.25	-5.72	5.68E-01
AJ516390	muc2 protein	0.4	-6.45	7.04E-01	2.33	0.11	1.57E-02	2.22	-1.03	2.20E-02
AJ626301	integrin beta 1 (fibronectin receptor beta)	0.24	-4.58	1.63E-01	0.33	0	1.72E-02	0.05	-6.34	8.50E-01
AJ624430	---NA---	-0.25	-6.1	5.35E-01	-0.49	-0.34	2.16E-02	0.7	1.19	4.35E-03
AJ626404	---NA---	0.69	6.24	3.67E-05	0.39	-1.19	4.49E-02	0.02	-6.54	9.83E-01
AJ516678	ependymin related protein 1	1.24	11.15	5.96E-07	0.6	-1.21	4.50E-02	1.15	9.52	1.24E-05
AJ624432	---NA---	0.6	2.5	8.80E-04	0.51	-1.37	5.08E-02	0.03	-6.55	9.90E-01
AJ625915	90-kda heat shock protein	-0.4	0.11	6.58E-03	-0.34	-2.13	9.03E-02	-0.12	-4.77	3.10E-01
AJ516677	arp2 actin-related protein 2 homolog	0.78	8.19	7.59E-06	0.34	-2.51	1.24E-01	0.74	7.79	3.77E-05
AJ624366	cornifelin	0.52	-5.48	3.35E-01	0.66	-2.57	1.27E-01	0.63	0.96	5.00E-03
AJ625700	ubiquitinisoform cra_a	0.59	7.21	1.56E-05	0.27	-2.6	1.27E-01	0.06	-6.41	8.90E-01
AJ516460	---NA---	0.83	13.42	1.17E-07	0.23	-2.78	1.49E-01	0.61	6.03	1.60E-04

AJ516903	loc562002 protein	0.83	2.79	6.87E-04	0.52	-2.85	1.57E-01	0.72	4.76	4.33E-04
AJ624665	universal stress protein	0.98	11.82	4.51E-07	0.34	-3.28	2.05E-01	0.43	0.61	6.53E-03
AJ516679	---NA---	1.02	7.66	1.11E-05	0.38	-3.58	2.46E-01	0.46	1.33	3.92E-03
AJ624844	ribosomal protein l18a	-0.28	1.3	2.45E-03	-0.15	-3.58	2.46E-01	-0.1	-4.71	2.94E-01
AJ625528	ribosomal protein l10a	-0.44	5.63	5.41E-05	-0.14	-3.62	2.50E-01	-0.04	-6.15	7.61E-01
AJ626296	ribosomal protein l12e	-0.37	0.38	5.38E-03	-0.24	-3.64	2.50E-01	-0.08	-4.87	3.27E-01
AJ623452	---NA---	0.55	5.95	4.45E-05	0.26	-3.68	2.53E-01	0.39	-0.24	1.23E-02
AJ623789	1-Precollagen-P	-0.35	3.25	4.65E-04	-0.18	-3.69	2.53E-01	-0.07	-6.54	9.83E-01
AJ516658	---NA---	0.57	2.25	1.08E-03	0.23	-3.79	2.68E-01	0.09	-3.3	1.11E-01
AJ516442	ependymin related protein 1	1.12	11.66	4.69E-07	0.36	-3.83	2.78E-01	0.81	6.84	8.62E-05
AJ516786	---NA---	0.84	7.74	1.07E-05	0.35	-3.93	2.97E-01	0.7	3.73	7.88E-04
AJ625055	---NA---	0.45	1.36	2.38E-03	0.26	-3.97	3.06E-01	0.38	-4.27	2.24E-01
AJ516624	---NA---	1.16	11.39	5.05E-07	0.35	-4.03	3.14E-01	0.93	7.84	3.77E-05
AJ625252	novel proteinvertebrate flavoprotein oxidore-ductase mical3	-0.06	-6.56	7.66E-01	-0.35	-4.05	3.17E-01	-0.8	0.46	7.55E-03
AJ516466	---NA---	0.47	5.98	4.42E-05	0.23	-4.11	3.28E-01	0.39	2.75	1.30E-03
AJ623776	---NA---	1.13	12.41	2.83E-07	0.37	-4.14	3.32E-01	1.58	13.96	1.66E-07
AJ625792	actin	-0.39	0.46	5.03E-03	-0.13	-4.16	3.32E-01	-0.51	1.34	3.92E-03
AJ624301	s2 ribosomal protein	-0.46	1.66	1.85E-03	-0.26	-4.35	3.78E-01	-0.23	-5.68	5.57E-01
AJ624090	neural precursor celldevelopmentally down regulated 9	-0.28	0.03	6.71E-03	-0.15	-4.42	3.87E-01	0	-5.75	5.79E-01
AJ624338	carboxypeptidase b	0.11	-5.55	3.54E-01	0.31	-4.43	3.90E-01	0.67	0.36	7.94E-03
AJ625065	---NA---	0.12	-6.12	5.47E-01	0.18	-4.53	4.12E-01	-0.29	0.94	5.03E-03
AJ516441	smc hinge domain containing 1	0.59	8.49	6.20E-06	0.13	-4.56	4.19E-01	0.33	3.46	8.19E-04
AJ626073	---NA---	0.47	0.29	5.64E-03	0.24	-4.56	4.19E-01	-0.1	-6.39	8.80E-01
AJ624309	paps synthetase	2.45	22.94	3.08E-11	0.28	-4.63	4.36E-01	2.68	24.3	2.10E-12
AJ516482	arginine kinase	0.53	2.47	8.91E-04	0.27	-4.66	4.39E-01	0.28	-2.16	5.11E-02
AJ624444	fibrinolytic protease 0	0.41	0.07	6.66E-03	0.26	-4.72	4.57E-01	-0.07	-6.37	8.69E-01
AJ625533	---NA---	1.22	0.57	4.65E-03	0.54	-4.83	4.84E-01	1.68	8.61	2.40E-05
AJ516578	cathepsin l	0.35	0.2	6.09E-03	0.16	-4.84	4.85E-01	-0.22	-5.63	5.43E-01
AJ516465	---NA---	0.65	8.16	7.59E-06	0.22	-4.85	4.86E-01	0.57	5.11	3.54E-04

AJ625531	serine protease inhibitor-1l	-0.41	1.5	2.08E-03	-0.2	-4.87	4.86E-01	-0.19	-5.07	3.72E-01
AJ625862	tmsb4x protein	-0.77	6.55	2.88E-05	-0.23	-4.87	4.86E-01	-0.24	-5.39	4.59E-01
AJ625268	ferritin	0.66	9.91	1.94E-06	-0.11	-5.01	5.30E-01	0.44	1.74	3.02E-03
AJ516425	---NA---	0.41	3.76	2.99E-04	0.10	-5.13	5.61E-01	0.29	-0.85	1.91E-02
AJ516652	---NA---	0.72	2.78	6.87E-04	0.29	-5.18	5.76E-01	0.71	-0.31	1.28E-02
AJ624341	myc homolog	0.09	-5.06	2.35E-01	0.08	-5.25	6.10E-01	0.84	6	1.60E-04
AJ623482	period clock protein	0.39	-2.23	3.05E-02	-0.07	-5.34	6.47E-01	1.05	3.38	8.19E-04
AJ624303	pleiotrophin-like protein	-0.24	-6.63	7.97E-01	-0.15	-5.41	6.63E-01	0.46	1.04	4.66E-03
AJ626010	---NA---	-0.49	2.5	8.80E-04	-0.15	-5.49	6.82E-01	-0.31	-3.73	1.51E-01
AJ625816	myna_mytgamyticin-a precursor	0.39	2.21	1.11E-03	0.16	-5.5	6.88E-01	0.32	3.69	7.88E-04
AJ625311	---NA---	-1.11	5.82	4.70E-05	-0.17	-5.51	6.89E-01	-0.05	-4.49	2.55E-01
AJ624869	cathepsin l	0.81	7.31	1.52E-05	0.27	-5.52	6.95E-01	0.59	-2.24	5.40E-02
AJ624678	---NA---	-0.32	-6.42	6.90E-01	-0.05	-5.53	6.97E-01	0.79	4.12	7.23E-04
AJ623334	---NA---	-0.55	2.83	6.79E-04	0.30	-5.54	7.00E-01	-0.16	-3.61	1.40E-01
AJ623465	---NA---	-0.09	-6.81	9.29E-01	-0.17	-5.55	7.05E-01	0.69	0.4	7.88E-03
AJ625323	loc562304 protein	-0.1	-6.27	6.13E-01	-0.17	-5.56	7.05E-01	-0.43	0.84	5.35E-03
AJ624482	developmentally-regulated vdg3	-0.17	-6.22	5.86E-01	0.01	-5.6	7.27E-01	0.43	1.13	4.51E-03
AJ624274	---NA---	0.16	-3.03	5.18E-02	0.04	-5.63	7.37E-01	0.4	1.75	3.02E-03
AJ516483	---NA---	0.74	9.28	3.16E-06	0.11	-5.65	7.37E-01	0.47	2.95	1.21E-03
AJ516456	---NA---	0.47	4.15	2.11E-04	0.10	-5.68	7.38E-01	0.29	-0.57	1.53E-02
not in database	---NA---	0.51	-0.52	9.58E-03	-0.20	-5.69	7.38E-01	0.9	2.14	2.17E-03
AJ623787	---NA---	-0.32	0.05	6.66E-03	-0.13	-5.69	7.38E-01	-0.09	-5.29	4.23E-01
AJ516642	---NA---	0.46	0.05	6.66E-03	0.17	-5.71	7.40E-01	0.5	0.35	7.95E-03
AJ625973	---NA---	-0.54	-0.96	1.32E-02	0.20	-5.78	7.65E-01	-0.56	1.07	4.66E-03
AJ623757	---NA---	-0.38	1.07	3.03E-03	-0.11	-5.79	7.65E-01	-0.09	-5.23	4.04E-01
AJ623726	ubiquitinisoform cra_a	0.46	1.34	2.39E-03	0.21	-5.84	7.81E-01	0.45	-1.81	4.04E-02
AJ624738	---NA---	-0.06	-6.85	9.70E-01	0.06	-5.87	7.91E-01	0.33	0.69	6.12E-03
AJ623481	ependymin related protein 1	1.75	6.28	3.65E-05	0.35	-5.91	8.12E-01	1.3	3.58	8.03E-04
AJ625443	---NA---	0.5	0.88	3.53E-03	-0.05	-5.95	8.23E-01	0.78	4.43	5.63E-04
AJ623651	---NA---	0.1	-6.86	9.93E-01	-0.09	-5.96	8.25E-01	0.87	0.09	9.72E-03

AJ625981	developmentally-regulated vdg3	-0.03	-6.5	7.30E-01	-0.03	-5.97	8.25E-01	0.55	3.54	8.03E-04
AJ626032	rna bindinghomolog 2	0.56	5.89	4.57E-05	-0.11	-5.99	8.42E-01	0.17	-4.65	2.86E-01
AJ625142	glyceraldehyde-3-phosphate dehydrogenase	0.36	3.27	4.65E-04	0.06	-6	8.45E-01	0.11	-4.83	3.24E-01
AJ623429	developmentally-regulated vdg3	0.04	-6.01	5.02E-01	-0.06	-6	8.45E-01	0.61	1.66	3.12E-03
AJ516426	---NA---	0.26	-0.45	8.99E-03	0.07	-6.01	8.48E-01	0.34	0.12	9.70E-03
AJ624759	---NA---	0.52	-3.62	8.24E-02	-0.06	-6.04	8.64E-01	-0.96	1.05	4.66E-03
AJ516427	---NA---	0.29	0.98	3.23E-03	0.08	-6.04	8.64E-01	0.21	-2.96	8.90E-02
AY484747	mItochondrial nadh dehydrogenase ND1	0.39	0.29	5.64E-03	0.03	-6.08	8.73E-01	-0.13	-6.42	8.96E-01
AJ623544	ependymin related protein-1 precursor	1.21	0.1	6.62E-03	0.25	-6.08	8.73E-01	0.66	-3	9.12E-02
AJ625582	---NA---	0.57	0.54	4.67E-03	0.11	-6.09	8.76E-01	-0.16	-6.56	9.99E-01
AJ625516	testicular haploid expressed gene	-0.4	3.4	4.19E-04	-0.02	-6.13	8.92E-01	0.05	-6.5	9.60E-01
AJ624336	---NA---	0.07	-5.48	3.36E-01	0.09	-6.14	9.00E-01	0.76	3.85	7.88E-04
AJ516474	tyra protein	1.05	8.13	7.59E-06	-0.08	-6.17	9.08E-01	0.67	2.76	1.30E-03
AJ516512	---NA---	0.07	-6.86	9.93E-01	0.09	-6.18	9.10E-01	-0.39	0.92	5.03E-03
AJ626213	glucose dehydrogenase	0.13	-5.81	4.35E-01	-0.17	-6.18	9.10E-01	-0.99	1.49	3.57E-03
AJ625131	microsomal glutathione s-transferase 3	0.4	-2.64	4.03E-02	-0.03	-6.19	9.16E-01	0.6	2.85	1.29E-03
AJ624361	matrilin-4-like protein	2.01	4.56	1.47E-04	-0.48	-6.21	9.19E-01	-1.25	-4.51	2.57E-01
AJ625133	O-methyltransferase family 3	0.76	14.17	6.53E-08	-0.09	-6.22	9.20E-01	0.44	3.64	7.91E-04
AJ625829	gram negative bacteria binding protein 2	-0.38	-3.91	9.75E-02	0	-6.3	9.56E-01	0.62	5.04	3.54E-04
AJ516802	ependymin related protein 1	0.58	5.18	8.06E-05	0.09	-6.36	9.71E-01	0.42	-1.75	3.93E-02
not in database1	gi|21703762|ref|NP_663355.1| similar to dendritic cell protein [Mus musculus]	1.41	7.19	1.56E-05	-0.19	-6.36	9.71E-01	-0.51	-4.87	3.27E-01
AJ626069	collagen type xii alpha 1	2.09	4.01	2.36E-04	-0.34	-6.41	9.75E-01	-0.84	-5.91	6.45E-01
AJ623462	---NA---	0.25	-3.61	8.20E-02	0.21	-6.42	9.80E-01	0.54	1.56	3.39E-03
AJ624753	juvenile hormone diol kinase	0.62	1.03	3.12E-03	-0.06	-6.42	9.80E-01	-0.26	-6.53	9.74E-01
AJ516379	---NA---	0.66	-1.25	1.50E-02	-0.01	-6.42	9.80E-01	0.91	3.4	8.19E-04
AJ516400	---NA---	0.02	-6.17	5.63E-01	-0.03	-6.42	9.80E-01	0.56	2.25	2.02E-03
AJ625256	matrilinisoform cra_b	2.42	5.8	4.70E-05	-0.35	-6.43	9.80E-01	-1.27	-4.46	2.52E-01
AJ623548	---NA---	0.15	-6.54	7.58E-01	0.09	-6.45	9.91E-01	0.7	3.36	8.19E-04
AJ516688	---NA---	-0.01	-6.57	7.66E-01	0.16	-6.46	9.94E-01	-0.36	0.15	9.55E-03

AJ624882	ferritin	0.75	-0.36	8.47E-03	0.09	-6.47	9.94E-01	0.59	0.36	7.94E-03
AJ623460	gram negative bacteria binding protein 2	-0.29	-5.78	4.28E-01	0.1	-6.47	9.94E-01	0.86	3.81	7.88E-04
AJ624926	small heat shock protein p26	0.81	0.55	4.67E-03	0.02	-6.47	9.95E-01	0.03	-5.79	5.96E-01
AJ625307	proteasomealpha type 5	0	-6.83	9.54E-01	0.04	-6.47	9.96E-01	-0.33	0.1	9.72E-03
AJ625629	---NA---	0.43	1.88	1.53E-03	0.09	-6.48	9.96E-01	0.54	-0.05	1.08E-02
not in database2	---NA---	-0.21	-3.85	9.39E-02	0.08	-6.48	9.96E-01	-0.31	1.22	4.32E-03
AJ625481	---NA---	0.65	-4.68	1.77E-01	0.22	-6.48	9.96E-01	1.22	3.77	7.88E-04

For each experimental condition (CHP, E_2, CHP/E_2) the embl gene ID (Gene) and the putative description assigned by means of the bioinformatic platform Blast2GO [48] are reported; M = log2 gene relative expression level; B = empirical Bayes log odd; Adj P = adjusted p value according to [64]. A gene was considered differentially expressed when a B>0 value was obtained according to the empyrical Bayes B-statistics [65]. B values lower that 0 are showed in red.
