# Supplementary material for: The Organophosphate Chlorpyrifos Interferes with the Responses to 17β-Estradiol in the Digestive Gland of the Marine Mussel Mytilus galloprovincialis
Source: PLoS One. 2011 May 20;6(5):e19803. doi: 10.1371/journal.pone.0019803 (PMC3098840; doi:10.1371/journal.pone.0019803)
Supplement: Table S2 — Supplementary information to Fig. 4 . Gene ID, gene description, expression trend of sequences reported in Fig. 4 are reported. (PDF) [file pone.0019803.s002.pdf]

Table S2. Supplementary information to Fig. 4.

Treatment	GO term	ID	Description	Expression trend	
CHP	regulation of multicellular organismal process	AJ623481	ependymin related protein 1	UP	
	regulation of multicellular organismal process	AJ624502	mam domain containing 2	DOWN	
	regulation of multicellular organismal process	AJ625531	serine protease inhibitor-1l	DOWN	*
	regulation of multicellular organismal process	AJ626032	rna bindinghomolog 2	UP	
	cell motility	AJ624059	calmodulin	UP	
	cell motility	AJ624502	mam domain containing 2	DOWN	
	cell motility	AJ625862	tmsb4x protein	DOWN	
	cell motility	AJ626032	rna bindinghomolog 2	UP	
	cell-matrix adhesion	AJ516442	ependymin related protein 1	UP	*
	cell-matrix adhesion	AJ516452	ependymin-related protein	UP	
	cell-matrix adhesion	AJ516678	ependymin related protein 1	UP	*
	cell-matrix adhesion	AJ516802	ependymin related protein 1	UP	
	cell-matrix adhesion	AJ516903	loc562002 protein	UP	*
	cell-matrix adhesion	AJ623481	ependymin related protein 1	UP	
	cell-matrix adhesion	AJ623544	ependymin related protein-1 precursor	UP	
	response to biotic stimulus	AJ624926	small heat shock protein p26	UP	
	response to biotic stimulus	AJ625816	myna_mytgamyticin-a precursor	UP	*
	response to biotic stimulus	AJ625915	90-kda heat shock protein	DOWN	
	response to biotic stimulus	AJ625974	heat shock protein 90	DOWN	
E2	cellular process	Aj626301	integrin beta 1 (fibronectin receptor beta)	UP	
	cellular process	Aj626667	cystathionine beta-synthase	UP	
	cellular process	AJ624059	calmodulin	UP	
	cellular process	AJ624502	mam domain containing 2	DOWN	
	cellular process	AJ624363	mam domain containing	DOWN	
	lipid metabolic process	AJ623364	Phospholipase A1 member i	UP	
	lipid metabolic process	AJ624495	gm2 ganglioside activator protein	UP	
	lipid metabolic process	AJ624509	phospholipasegroup xiia	UP	
	lipid metabolic process	AJ626301	integrin beta 1 (fibronectin receptor beta)	UP	
CHP/E2	cellular metabolic process	AJ516903	loc562002 protein	UP	*
	cellular metabolic process	Aj624667	cystathionine beta-synthase	UP	*
	cellular metabolic process	AJ625816	myna_mytgamyticin-a precursor	UP	*
	cellular metabolic process	AJ624309	paps synthetase	UP	*
	cellular metabolic process	AJ625133	O-methyltransferase family 3	UP	*
	cell-matrix adhesion	AJ516442	ependymin related protein 1	UP	*
	cell-matrix adhesion	AJ516678	ependymin related protein 1	UP	*
	cell-matrix adhesion	AJ516903	loc562002 protein	UP	*
	cell-matrix adhesion	AJ623481	ependymin related protein 1	UP	*
	carbohydrate metabolic process	AJ516903	loc562002 protein	UP	*
	carbohydrate metabolic process	AJ624341	myc homolog	UP	
	carbohydrate metabolic process	AJ625131	microsomal glutathione s-transferase 3	UP	
	carbohydrate metabolic process	AJ625829	gram negative bacteria binding protein 2	UP	
	carbohydrate metabolic process	AJ626213	glucose dehydrogenase	DOWN	

Gene ID, gene description, expression trend of sequences reported in Fig. 4 are reported. * = differentially expressed genes in common between CHP and CHP/ E_2.
