# Supplementary figures and images for: The Organophosphate Chlorpyrifos Interferes with the Responses to 17β-Estradiol in the Digestive Gland of the Marine Mussel Mytilus galloprovincialis
Source: PLoS One. 2011 May 20;6(5):e19803. doi: 10.1371/journal.pone.0019803 (PMC3098840; doi:10.1371/journal.pone.0019803)

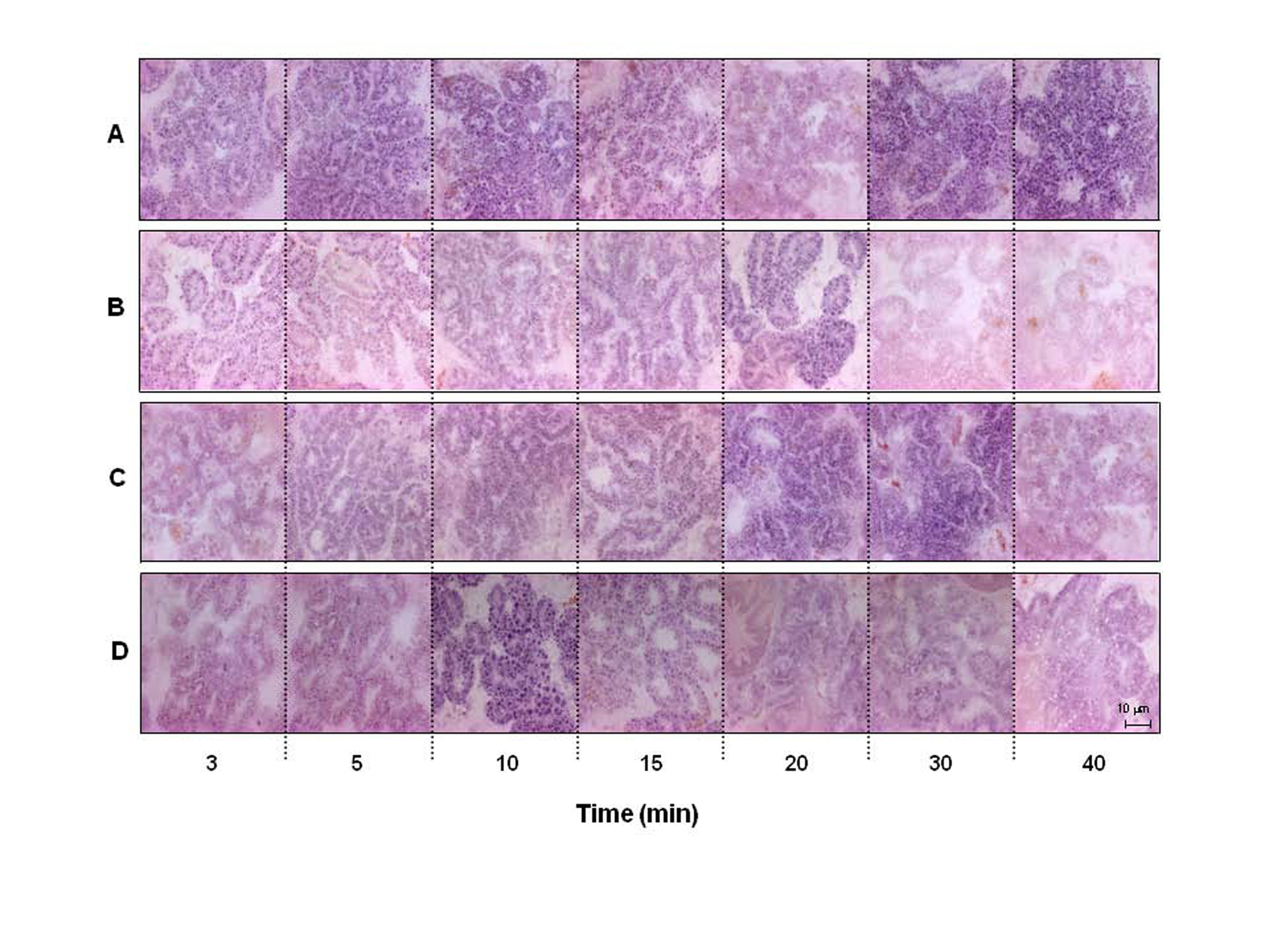

Supplement: Figure S1 — Determination of Lysosomal membrane stability (LMS) by assessment of latent lysosomal N-acetyl-508β-hexosaminidase activity in cryostat sections of frozen mussel digestive gland as described in [58] . Sections were pre-treated at pH 4.5 and 37°C for 3–40 minutes (3, 5, 10, 15, 20, 30, 40 minutes, respectively). Representative images of A = Control DMSO/EtOH; B = CHP; C = E2; D = CHP/E2, where maximal lysosomal staining intensity represents the labilization period. (Scale Bar = 10 µm). (TIF) [file pone.0019803.s003.tif]
